# Innovative Technological Approach to Ebola Virus Disease Outbreak Response in Nigeria Using the Open Data Kit and Form Hub Technology

**DOI:** 10.1371/journal.pone.0131000

**Published:** 2015-06-26

**Authors:** Daniel Tom-Aba, Adeniyi Olaleye, Adebola Tolulope Olayinka, Patrick Nguku, Ndadilnasiya Waziri, Peter Adewuyi, Olawunmi Adeoye, Saliu Oladele, Aderonke Adeseye, Olukayode Oguntimehin, Faisal Shuaib

**Affiliations:** 1 African Field Epidemiology Network, Abuja Office, Abuja, Nigeria; 2 UNICEF Office, Lagos, Nigeria; 3 Nigeria Field Epidemiology and Laboratory Training Programme, Abuja, Nigeria; 4 Ahmadu Bello University, Zaria, Nigeria; 5 WHO Country Office, Abuja, Nigeria; 6 Lagos State Ministry of Health, Lagos, Nigeria; 7 Lagos State Primary Health Care Board, Lagos, Nigeria; 8 Federal Ministry of Health, Abuja, Nigeria; University of Louisville School of Medicine, UNITED STATES

## Abstract

The recent outbreak of Ebola Virus Disease (EVD) in West Africa has ravaged many lives. Effective containment of this outbreak relies on prompt and effective coordination and communication across various interventions; early detection and response being critical to successful control. The use of information and communications technology (ICT) in active surveillance has proved to be effective but its use in Ebola outbreak response has been limited. Due to the need for timeliness in reporting and communication for early discovery of new EVD cases and promptness in response; it became imperative to empower the response team members with technologies and solutions which would enable smooth and rapid data flow. The Open Data Kit and Form Hub technology were used in combination with the Dashboard technology and ArcGIS mapping for follow up of contacts, identification of cases, case investigation and management and also for strategic planning during the response. A remarkable improvement was recorded in the reporting of daily follow-up of contacts after the deployment of the integrated real time technology. The turnaround time between identification of symptomatic contacts and evacuation to the isolation facility and also for receipt of laboratory results was reduced and informed decisions could be taken by all concerned. Accountability in contact tracing was ensured by the use of a GPS enabled device. The use of innovative technologies in the response of the EVD outbreak in Nigeria contributed significantly to the prompt control of the outbreak and containment of the disease by providing a valuable platform for early warning and guiding early actions.

## Introduction

The recent outbreak of Ebola Virus Disease (EVD) in West Africa has been declared by the World Health Organization (WHO) as the world's deadliest international health emergency in the modern time.[1] It has been unique and unusual being unprecedented in size and geographical distribution affecting major cities and the first time cases were recorded in Nigeria. Effective containment of EVD relies on an approach that involves multiple interventions: case management, surveillance and contact tracing, communication and social mobilization. Timely transfer of information is central to effective coordination and communication across these interventions, early detection and early response which are key to a successful EVD control The use of active surveillance which consists of close supervision and systematic timely collection of vital signs and key clinical symptoms from contacts to EVD cases is critical to the effective control of EVD.[2] The, deployment, application and use of information technology (IT) is therefore imperative in offering a valuable platform for early warning and early response. With the recent outbreak, many countries are now investing in various technologies for early containment such as the installation of thermal scanners at airports. [2]

In the recent times, IT has penetrated the domain of public health in many countries and the potential for harnessing information technology for surveillance is evident [3]. The use of mobile phone technology to address health needs have been documented in many African countries. For example, SMS was used for reporting in a surveillance system in Madagascar,[4] hundreds of Ugandan health workers used Personal Digital Assistants (PDAs) to collect health data in the field.[5] While there is evidence of the use of IT in active surveillance and routine health information management system, the use of information and communications technology (ICT) or public health informatics in the fight against the Ebola Virus Disease has been limited. The outbreak of EVD in the two Nigerian cities of Lagos and Port Harcourt however presented unique challenges that necessitated the use of innovative IT. Lagos is a mega city with an estimated population of 21million people [6] while Port Harcourt is another large city in Nigeria. Heavy traffic within the cities made it almost impossible for the Contact tracers to submit daily follow up reports on the health status of over 800 Contacts who lived at different communities scattered across 23 Local Government Areas with 17 in Lagos and 6 in Port Harcourt. Specifically, the initial EVD response efforts in Nigeria were confronted with the following challenges:

1. Slow pace of manual entry of large volume of data from multiple sources using the Epi-Info database of viral hemorrhagic fevers developed by the US Centers for Disease Control on a single computer system, resulting in backlog of data.
2. Delay in receiving reports from the Contact Tracers when they identified onset of symptoms in contacts and or new contacts, causing delay in evacuation of new suspects and registration of new contacts.
3. High turn-around time for receipt of laboratory results leading to delays in confirmation of status of suspect cases on admission for prompt actions by the case management team.
4. The peculiar traffic situation in Lagos and Port Harcourt which led to a delay in collation of reports from different team members.
5. Delayed communication across response teams causing delays in taking immediate follow up actions and strategic decisions.

Due to the need for timeliness in reporting and communication for early discovery of new EVD cases and promptness in response; it became imperative to empower the response team members with technologies and solutions which would enable smooth and rapid data flow. The Strategy group of the Ebola Emergency Operations Committee had a discussion with the IT team and took a decision on deploying IT methods to overcome some of these challenges. Previous experience gained in using the ODK by members of the IT team during Polio surveys and enumeration of hard to reach areas in the country served as a leverage in deploying these techniques. Both ODK and Formhub are open-source and free which need just smartphones and cloud servers to digitalize data collection. This required the setting up of a real time system to ensure that the process is automated from data capture to data retrieval. The EEOC already had a server, funds from the EEOC bought the smartphones and tablets needed, the IT team had the knowledge and expertise and the technology was open source and free. This paper describes the innovative use of public health informatics tools to improve response to an Ebola virus disease outbreak in Nigeria.

## Materials and Methods

Two android base applications were the major technologies used—The Open Data Kit (ODK) (www.opendatakit.org) and Form Hub Technology (www.formhub.org). Supporting technologies were the Dashboard technology and ArcGIS mapping. The contact listing form, contact follow up form, laboratory investigation request form and case investigation forms were created using the extensible markup language (xml) and eHealth Ebola Sense android app developed for 21 day follow up (https://play.google.com/store/apps/details?id=com.ehealthafrica.senseebola).

**Fig 1:**
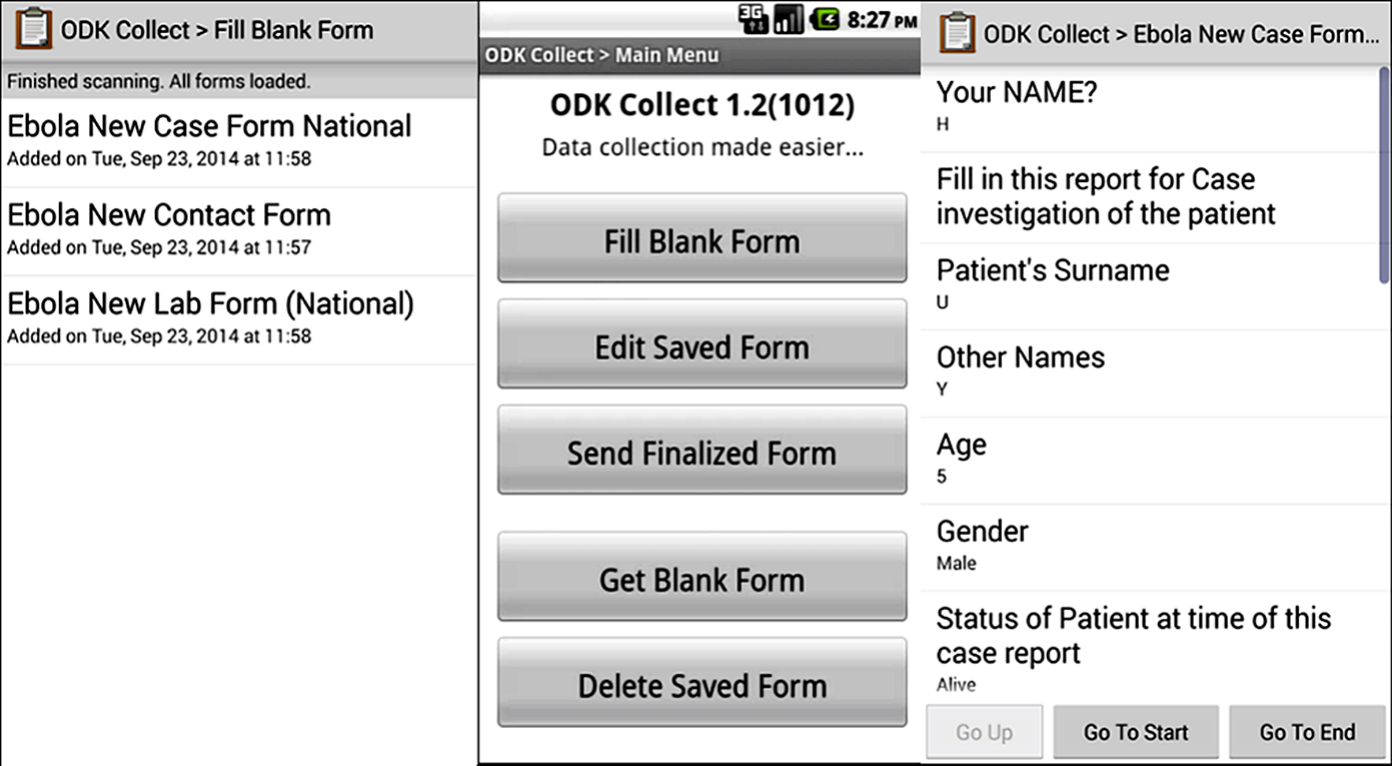
Screen shot of ODK collect App on mobile phones and ehealth follow up Ebola sense.

These forms were adapted from the WHO guidelines for Ebola Virus Disease Outbreak Response. The forms were uploaded on the Form Hub server and downloaded to android devices (smartphones or tablets) using the ODK collect application (Fig 1). Smartphones were used for contact tracing while tablets were used for laboratory and case investigations. Each contact tracing team was equipped with a smartphone and the paper forms for contact listing and contact follow up while the laboratory and case management teams were given the tablets.

The initial contact listing was done using the paper forms but subsequently as new contacts were identified, the listing was done using both the paper and the ODK forms. Each day as contacts were being monitored for symptoms of EVD, the temperature reading and presence or absence of clinical signs or symptoms of EVD were recorded using the smartphones as well as the GPS coordinates of the place of visit. Once the information was entered in the ODK form on the phone, the finalized form was sent automatically to the Form Hub server that housed information on all contacts in a database.

**Fig 2:**
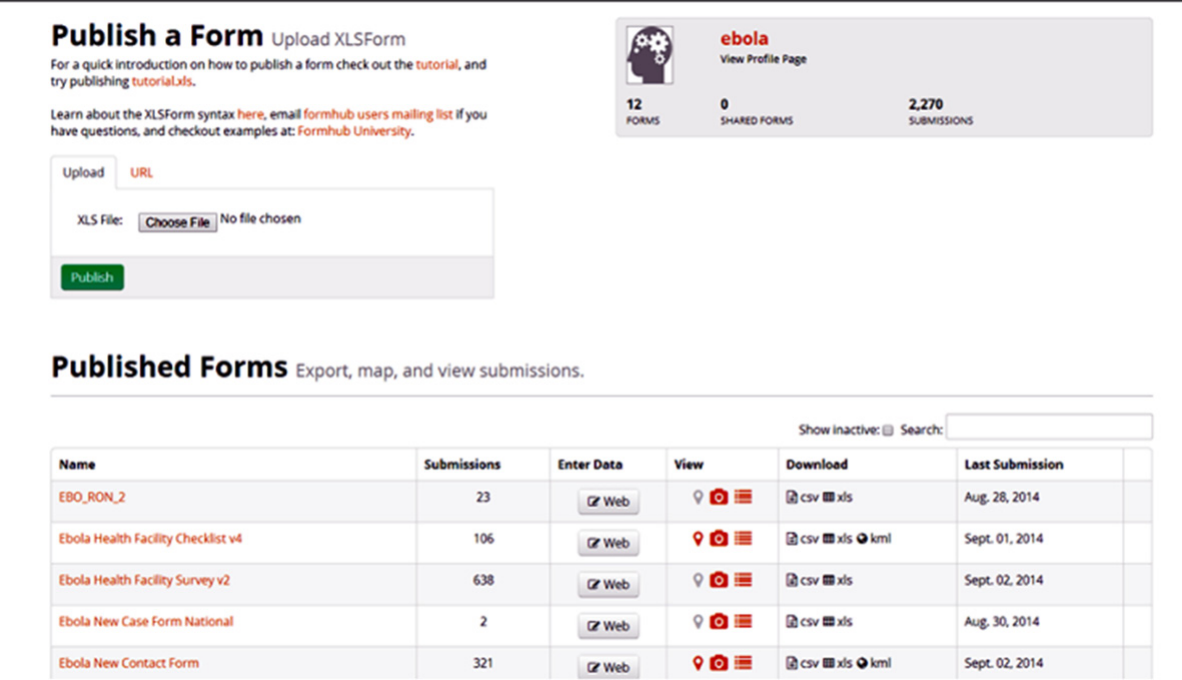
Screen shot of Form Hub page.

If a contact had a temperature of ≥38°C and/or any of the indicated EVD symptoms the information was recorded in the phone, an alert was immediately sent to the server using a Cron Job Script [7] using a Cron job command tab (http://www.thesitewizard.com/general/set-cron-job.shtml) which generates a red highlight on the contact’s information on the dashboard (Fig 2). In addition, the system was programmed to send a text message using the short message service (SMS) to the Incident manager, Deputy Incident Manager and Team leads of Contact tracing and Case Management in order to activate an evacuation plan.

Similarly, for the laboratory investigation request and case Investigation forms uploaded to the tablets, information entered was automatically sent to the case management team, the strategy group and data management team. This was used for real time notification of laboratory results, determination of infection status of the suspects and immediate actions to be taken on suspects. The Case Management team sent daily clinical reports through the hub server to the Strategy Group. The information sent could be visualized on a screen as a dashboard.

The interaction between the form hub server where all data was based and the web dashboard was controlled automatically by a Cron Job Script. All data entry points were either through the mobile phones or through the web forms on the form hub server. All new contacts registered by the contact tracers, once verified by an administrator were automatically moved to the 21 days follow up database. The contacts under follow-up were monitored with the mobile app called eHealth Sense.

The eHealth Sense works like an automated search engine which searches for each contact and makes pop up suggestions as you type in names of contact tracers. Once a contact is selected, it allows the daily follow up to be done for that day, the status of the follow-up was automatically displayed real time on the dashboard.

The operational research, point of entry and social mobilization teams also used the ODK for data collection for various activities during the Nigerian EVD outbreak response. Members of each team were trained on the use of these applications and devices before they were sent to the field.

### Hardware and Software requirement for the Technology

1. Laptops
2. Tablets
3. Good Android Phones (Jelly bean Android OS) Large touch screen or QWERTY Keys.
4. High speed Internet
5. Good Mobile data plans.
6. eHealth Sense mobile application
7. ODK Collect Software
8. Viewing device such as television for viewing real time data and maps

### Description of the Technologies Used

1 **Open Data Kit (ODK) Collect Application**. The ODK Collect application was used in creating respective data collection forms (interfaces) which enable users enter data or edit saved data on their phones/tablets. The entered data are then submitted on finalization. The geo-coordinates were automatically embedded in the data during submission.

Seven set of forms were created for the ODK collect. These were the New Contact Form, Contact Follow-Up Form, Laboratory Request/Result Form, Case Investigation Form, Social Mobilization Form, Point of Entry Screening Form and Health Facility Survey Form.

**Fig 3:**
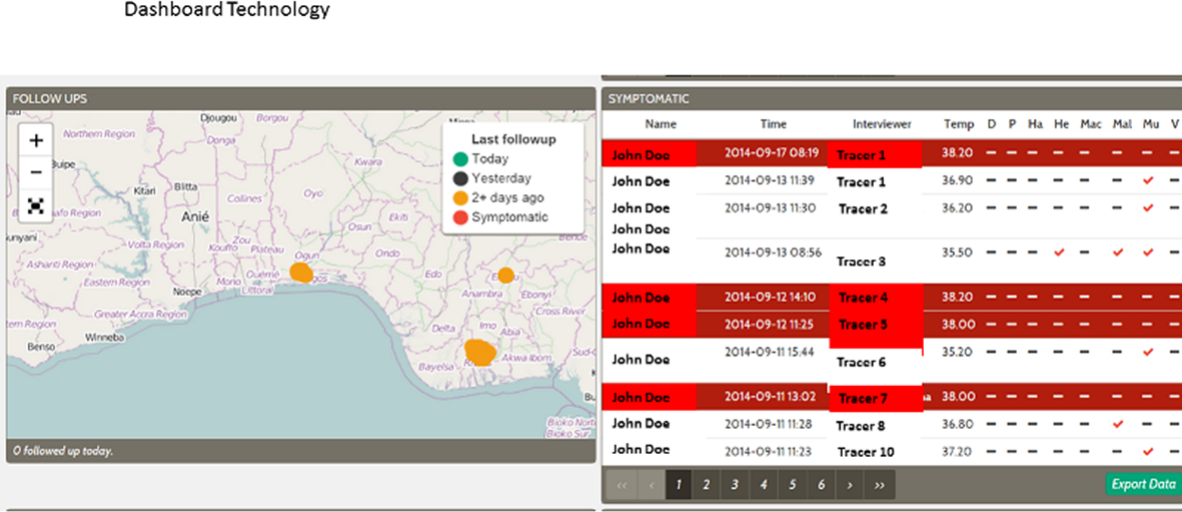
Screen shot of web dashboard built with python and git hub using PLSQL Database.

2 **The Form Hub Technology**. The hub technology forms were built based on the hard paper forms being used to collect data from the field. When new cases are identified, the details are entered into a new case form on the mobile phone by the contact tracer which is sent directly to the form hub server “Fig 2” provided the phones have good internet connection with data plans on them. If for any reason, the location is out of internet connection then the data collected are temporarily stored on the phones. When the phones are returned back at the end of the day to the control center, the phones are automatically or manually connected to the Wi-Fi then all the data synchronizes and are added to the form hub server.
3 **Dashboard Technology**. The Dashboard comprises of a segmented output, which focuses on providing evidence-based information to support management in taking prompt decisions. An internet-capable television is used to display the status of contacts followed-up daily, the interviewers and their performance in terms of proportion of contacts followed up “Fig 3”. It also displays or flags-off symptomatic contacts, displays maps showing locations of contacts followed-up in the last two days. Lab results of suspected or confirmed cases are also displayed in one of the segments, of the dashboard.
4 **ArcGIS Mapping**. Locations of identified contacts and cases were mapped using the coordinates recorded on the ODK forms and integrated to show the distribution and clustering of contacts and cases within the cities using ArcGIS software. This informed areas to be targeted for social mobilization, sensitization and awareness creation by the social mobilization teams.

## Results, Discussions and Conclusions

**Fig 4:**
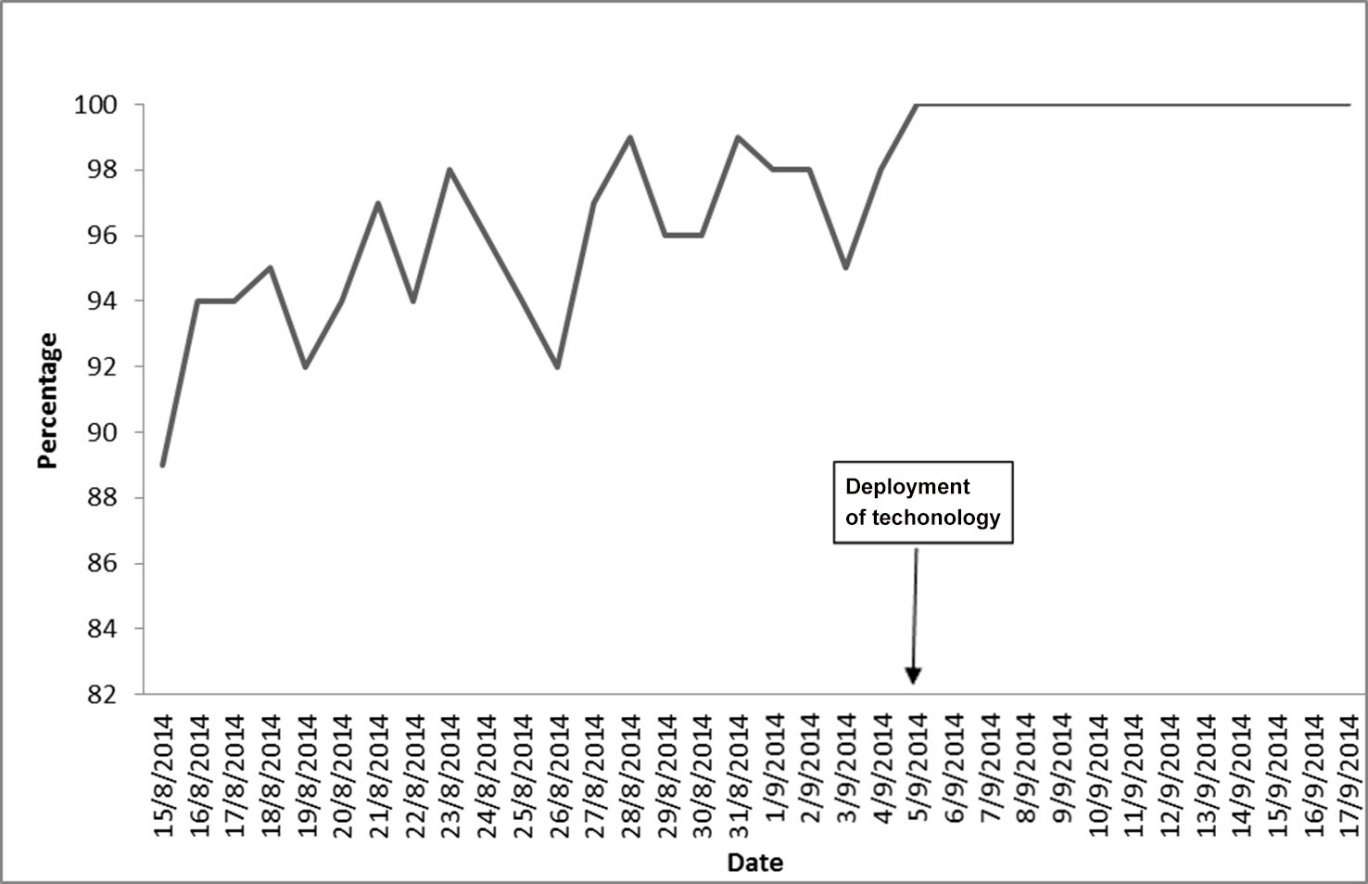
Trend of contact follow-up before and after deployment of Mobile application.

Improvement was recorded in the reporting of daily follow-up of contacts after the deployment of the integrated real time technology. Fig 4 shows that daily reporting of contact follow up before 5pm was less than 100% in August 2014 before the deployment of mobile technology; but from 5 September follow-up reports attained 100% and stabilized thereafter. However it is difficult to attribute this improvement to the use of the technology alone as other factors could have contributed like tracers getting more experienced at the job and number of contacts to be followed up reducing towards the end of the epidemic.

The electronic data collection and alert system reduced the turnaround time between identification of symptomatic contacts and evacuation to the isolation facility from between 3 to 6 hours to an hour. This aided prompt evacuation of symptomatic contacts to reduce or stop further transmission of the virus in the community.

Also, the turnaround time for laboratory results was considerably reduced. This aided the immediate discharge of suspected cases with negative laboratory results from the suspect ward as well as prompt movement of suspected cases with positive laboratory results to the confirmed ward and commencement of supportive treatment.

In addition, the Incident Manager and strategy group had timely and complete information required to make informed decisions on all issues. Deployment of the integrated technology also provided other benefits such as simultaneous multiple data entry by different persons into a single database, thereby eliminating the dependency on a single data entry clerk. It introduced accountability in contact tracing exercise thereby ensuring that all contacts were seen physically in their homes because of the use of a GPS enabled device. It also facilitated daily collation of reports from different team members for daily evening review meetings and daily situation report.

The deployment of the technology was not without some challenges and these were the initial costs of setting up the required technology (getting the phones and tablets, preloading them with the required forms), the need for trained personnel (most of our contact tracers were residents of the Nigeria Field Epidemiology and Laboratory Training Programme and other public health professionals) many had participated in polio surveys, some had used the ODK before and most knew how to use smartphones and tablets) and the need to have highly effective internet connections to run the applications. Also the integration of Epi-info VHF with form hub technology to align same data structure across the system posed some challenges.

The use of innovative technologies in the response of the EVD outbreak in Nigeria contributed significantly to the prompt control of the outbreak and containment of the disease by guiding the prompt evacuation of symptomatic contacts from the community to the isolation facility thereby reducing contact with others and reducing the risk of transmission of the virus.
